# Supplementary material for: Easily Implementable Dietary Improvement Using Recipes: Analytical Method Applied to a Specific Region in Japan
Source: Nutrients. 2025 May 8;17(10):1614. doi: 10.3390/nu17101614 (PMC12114614; doi:10.3390/nu17101614)
Supplement: Supplementary file 1 [file nutrients-17-01614-s001.zip › nutrients-3574009-supplementary.pdf]

Supplementary materials

Figure S1. Examples of θ, η, and φ for a standardized nutrient content vector z in three dimensions.

The figure below illustrates the properties indicated by narrow complementarity θ, quantitative effect η, and symmetry concerning quantity φ for a standardized nutrient content vector z in three dimensions. Although the three axes appear to be orthogonal in the figure, they are actually oblique because the vector z is standardized using the standard deviation matrix S of the nutrient content vector x.

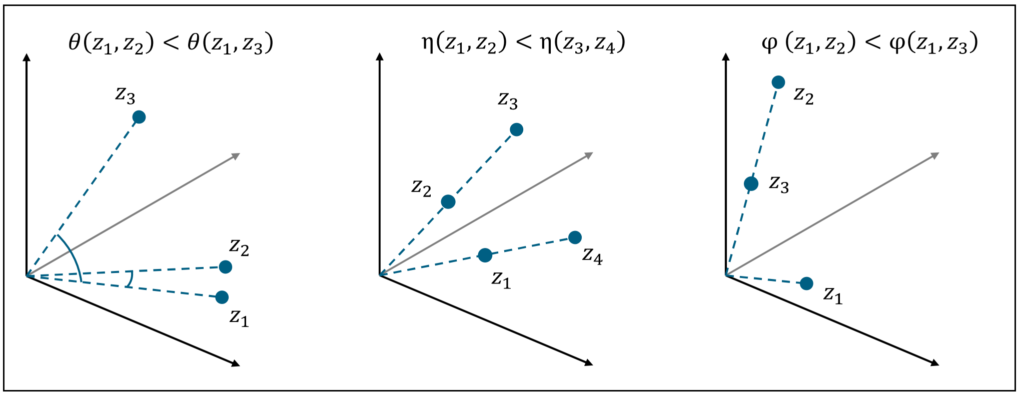

Note S1. The relationship between Mahalanobis distance and complementarity (θ, η, φ).

As shown in the following equation, the Mahalanobis distance $d(x_i, x_j)$ is a function of three variables (θ, η, φ).

$$d(x_i, x_j) \equiv (x_i - x_j)' \widehat{\mathrm{Var}(x)}^{-1} (x_i - x_j) = |z_i|^2 + |z_j|^2 - 2z_i' z_j = \eta^2 \left(1 - 2\sin\frac{\pi}{4}\varphi\cos\frac{\pi}{4}\varphi\cos\theta\right)$$

In its domain, the Mahalanobis distance d is an increasing function of θ and a non-decreasing function of η. The Mahalanobis distance d is an increasing function of φ in the range $\theta > \pi/2$, but a decreasing function of φ in the range $\theta < \pi/2$. The former range represents the data range where combinations of foods exhibit exceptional values in relation to the positive correlation, if any, between the nutrients that constitute the nutrient content vector. When measuring the complementarity of nutrients using the Mahalanobis distance, it effectively captures the narrow sense of complementarity θ and quantitative effect η, but fails to adequately reflect the symmetry of quantity φ. Even if the Mahalanobis distance between two foods with asymmetric nutrient quantities is large, it cannot account for the situation where the smaller food "free-rides" on the larger food. This aspect can be considered a drawback of the Mahalanobis distance in light of the analytical purposes of this paper.

Note S2. A numerical example illustrating the calculation of nutrient complementarity:

The calculation procedure for nutrient complementarity concerning three selected nutrients—calcium (Ca), niacin, and vitamin B6—is presented below for the group of 82 food items (Table A1) for which nutrient data per 100-yen equivalent is available.

Step 1. Sequentially compute the following for the nutrient contents of the food items being analyzed: (1) the covariance matrix $\mathrm{Var}(x)$, (2) the standard deviation matrix$S \equiv \mathrm{Var}(x)^{1/2}$ (The standard deviation matrix can be calculated using, for example, the sqrtm function in Python), and (3) the inverse of the standard deviation matrix S^{-1}. The calculation results are shown below (additionally, the correlation coefficient matrix is provided for reference). The units of nutrient content per 100-yen equivalent are as follows: Ca and vitamin B6 are in mg, while niacin is in niacin equivalent mg. Among the 82 analyzed food items, the variance in Ca content is larger, whereas the variance in vitamin B6 content is smaller. There is a negative

correlation between the content of Ca and niacin, and a positive correlation between the content of niacin and vitamin B6.

Var(x)

	Ca	niacin	vitamin B6
Ca	9492.4	-51.1	0.98
niacin	-51.1	6.78	0.14
vitamin B6	0.98	0.14	0.02

Corr(x)

	Ca	niacin	vitamin B6
Ca	1.00	-0.20	0.07
niacin	-0.20	1.00	0.35
vitamin B6	0.07	0.35	1.00

S

	Ca	niacin	vitamin B6
Ca	97.43	-0.51	0.010
niacin	-0.51	2.55	0.054
vitamin B6	0.010	0.054	0.143

S^{-1}

	Ca	niacin	vitamin B6
Ca	0.010	0.002	-0.002
niacin	0.002	0.395	-0.150
vitamin B6	-0.002	-0.150	7.058

Step 2. For each food item in the analyzed group, calculate the standardized vector (z) by multiplying the inverse of the standard deviation matrix on the left side of the nutrient content vector (x). The values of the nutrient content vector (x) per 100-yen equivalent and the standardized vector (z) for the analyzed food items are shown below.

Food id	Food	Ca [mg]	Niacin [mgNE]	Vitamin B6 [mg]	z(Ca)	z(Niacin)	z(Vitamin B6)
1	beef	1.5	1.8	0.15	0.02	0.68	0.77
2	pork	1.8	2.7	0.14	0.02	1.1	0.61
3	chicken	4.1	4.1	0.22	0.05	1.6	0.91
4	liver	3.9	7.2	0.47	0.05	2.8	2.3
5	tofu	358.0	0.77	0.19	3.7	1.0	0.69
6	ham	1.8	3.3	0.13	0.03	1.3	0.39
7	sausage	3.2	1.9	0.08	0.04	0.76	0.24
8	bacon	2.3	1.1	0.07	0.03	0.45	0.31
10	milk	574.0	0.52	0.16	5.9	1.4	0.15
11	egg	209.1	0.45	0.41	2.1	0.56	2.5
12	cheeses	329.8	0.05	0.01	3.4	0.71	-0.48
13	yogurt	353.6	0.29	0.06	3.6	0.85	-0.17
14	salted fish	7.7	1.8	0.14	0.08	0.70	0.68
15	canned tuna	2.8	6.2	0.18	0.04	2.4	0.36
16	salmon & trout	4.2	2.0	0.19	0.05	0.78	1.1
17	tunas & bonito	2.0	4.8	0.18	0.03	1.9	0.54
18	amberjack	6.2	4.8	0.23	0.07	1.9	0.87
19	pollack & flatfish	14.7	0.77	0.04	0.15	0.33	0.17
20	horse mackerel & sardine	31.2	2.8	0.18	0.33	1.2	0.77
21	pacific saury & mackerel	12.0	6.7	0.36	0.14	2.6	1.5
22	shirasuboshi	66.8	0.62	0.01	0.69	0.38	-0.11
23	cod roe & salmon roe	5.3	11.1	0.06	0.08	4.4	-1.28
25	squid	6.6	2.4	0.13	0.07	0.95	0.52
26	octopus	6.3	0.87	0.03	0.07	0.35	0.06
27	shrimp	8.4	0.59	0.02	0.09	0.25	0.02
28	clam & corb shell	32.5	0.24	0.00	0.33	0.16	-0.06
29	fish sausage	18.8	0.88	0.01	0.19	0.38	-0.07
30	boiled fish paste	16.7	0.33	0.01	0.17	0.17	-0.03
31	fried fish paste	68.5	0.57	0.02	0.70	0.37	-0.03
32	mandarin	23.4	0.33	0.07	0.24	0.17	0.39
33	other oranges	34.1	0.65	0.11	0.35	0.31	0.65
34	apple	6.0	0.15	0.06	0.06	0.06	0.39
35	strawberry	8.5	0.20	0.02	0.09	0.09	0.10
36	peach	3.2	0.49	0.02	0.03	0.20	0.04
37	pears	2.5	0.25	0.03	0.03	0.10	0.14
38	kiwi fruit	24.8	0.29	0.10	0.26	0.15	0.66
40	banana	12.2	1.4	0.77	0.13	0.47	5.2
41	pickled radish	29.2	0.18	0.02	0.30	0.13	0.06
42	pickled plum	10.3	0.04	0.01	0.11	0.04	0.07
43	pickled chinese cabbage	50.2	0.51	0.10	0.52	0.29	0.57
45	carrot	57.7	1.6	0.22	0.60	0.70	1.2

Food id	Food	Ca [mg]	Niacin [mgNE]	Vitamin B6 [mg]	z(Ca)	z(Niacin)	z(Vitamin B6)
46	spinach	38.3	0.47	0.11	0.39	0.25	0.64
47	pumpkin	30.6	3.1	0.45	0.32	1.2	2.7
48	cabbage	172.2	0.80	0.44	1.8	0.61	2.7
49	radish	117.2	1.0	0.25	1.2	0.61	1.5
50	green pepper	9.3	0.51	0.16	0.10	0.20	1.0
51	tomatoes	8.3	0.83	0.09	0.09	0.33	0.53
52	japanese leek	33.0	0.37	0.11	0.34	0.20	0.67
57	broccoli	46.6	0.93	0.28	0.48	0.42	1.8
58	onion	62.6	0.37	0.52	0.64	0.20	3.5
59	cucumber	42.0	0.32	0.08	0.43	0.20	0.46
60	eggplant	21.2	0.59	0.06	0.22	0.27	0.30
61	chinese cabbage	165.4	2.3	0.35	1.7	1.2	1.8
62	burdock	56.5	0.49	0.12	0.58	0.29	0.71
63	bean sprout	77.5	2.3	0.39	0.80	1.0	2.3
64	snap bean	16.3	0.20	0.02	0.17	0.11	0.11
65	lettuce	36.0	0.38	0.09	0.37	0.21	0.56
66	green asparagus	7.8	0.41	0.05	0.08	0.17	0.27
68	bread	46.8	2.3	0.06	0.49	1.0	0.03
69	japanese noodles (udon)	15.5	0.52	0.03	0.16	0.23	0.08
71	chinese noodles	33.6	0.34	0.00	0.35	0.20	-0.10
72	pasta	35.3	2.7	0.09	0.37	1.1	0.17
73	japanese noodles (somen)	37.4	1.2	0.00	0.39	0.57	-0.24
74	rice cake	4.3	0.29	0.04	0.04	0.12	0.25
75	cakes	10.4	0.06	0.02	0.11	0.04	0.08
76	biscuit cookie	14.4	0.43	0.03	0.15	0.20	0.12
77	chocolate	241.4	1.2	0.11	2.5	0.97	0.23
78	ice cream	75.7	0.05	0.01	0.78	0.18	-0.05
80	peanuts	42.6	14.5	0.41	0.47	5.8	0.65
83	fried tofu	265.8	0.17	0.06	2.7	0.61	-0.01
84	fermented soybeans	118.4	1.4	0.32	1.2	0.77	1.8
85	sweet potatoes	75.9	1.1	0.38	0.78	0.55	2.4
86	potatoes	10.4	3.9	0.52	0.11	1.5	3.1
87	taros	13.7	0.78	0.14	0.14	0.32	0.83
88	yams	22.8	0.54	0.12	0.23	0.24	0.74
89	konjac	121.5	0.00	0.06	1.2	0.25	0.21
90	shiitake mushroom	0.69	2.4	0.15	0.01	0.91	0.68
91	enoki mushroom	0.00	9.8	0.17	0.02	3.9	-0.25
92	shimeji mushroom	1.2	7.4	0.11	0.03	2.9	-0.34
93	seaweed wakame	63.3	0.10	0.00	0.65	0.17	-0.08
94	seaweed hijiki	66.1	0.00	0.00	0.68	0.14	-0.10
95	seaweed nori	22.0	0.94	0.05	0.23	0.41	0.15

Transforming vector x into vector z not only standardizes the variation in nutrient content but also has the following effect: when comparing combinations of nutrients with negative correlations in their content, such as Ca and niacin, with combinations showing positive correlations, such as niacin and vitamin B6, the differences between foods that contain relatively higher amounts of the first nutrient and lower amounts of

the second, and foods that contain relatively lower amounts of the first nutrient and higher amounts of the second, are weighted more heavily on the vitamin B6-niacin plane than on the niacin-Ca plane. This corresponds to a coordinate transformation caused by the matrix S^{-1}, in which the intersection angle between the Ca axis and the niacin axis becomes smaller than a right angle, while the intersection angle between the niacin axis and the vitamin B6 axis becomes larger than a right angle. For example, on the niacin-Ca plane, the coordinates of milk and cod roe/salmon roe before transformation are x1=(0.52, 574.0) and x2=(11.1, 5.3), while after transformation they are z1=(1.4, 5.9) and z2=(4.4, 0.08). On the vitamin B6-niacin plane, the coordinates of bananas and peanuts before transformation are x1=(0.77, 1.4) and x2=(0.41, 14.5), while after transformation they are z1=(5.2, 0.47) and z2=(0.65, 5.8). The differences between milk and cod roe/salmon roe on the niacin-Ca plane, and between bananas and peanuts on the vitamin B6-niacin plane, are adjusted by the standardization of the standard deviations of nutrient content and the effect of the transformation to the aforementioned oblique coordinate system.

Step 3. For all pairs of food items within the analyzed group, compute the nutrient complementarity indices θ, η, and φ based on their standardized vectors (z). Among the 3,321 combinations of two foods selected from 82 food items, the top 5 and bottom 5 results for the composite measure I are shown below.

food1	food2	∣z1∣	∣z2∣	η	φ	θ	η_cdf	φ_cdf	θ_cdf	I
banana	peanuts	5.26	5.81	7.83	0.94	1.37	0.9994	0.9425	0.9265	0.9265
onion	enoki mushroom	3.55	3.87	5.25	0.94	1.58	0.9211	0.9512	0.9904	0.9211
cheeses	enoki mushroom	3.50	3.87	5.22	0.94	1.35	0.9202	0.9407	0.9193	0.9193
liver	yogurt	3.57	3.73	5.16	0.97	1.41	0.9190	0.9720	0.9467	0.9190
yogurt	onion	3.73	3.55	5.15	0.97	1.43	0.9187	0.9696	0.9548	0.9187
…	…	…	…	…	…	…	…	…	…	…
strawberry	pickled plum	0.16	0.13	0.21	0.86	0.43	0.0006	0.8765	0.2252	0.0006
pickled plum	peanuts	0.13	5.81	5.81	0.03	1.16	0.9455	0.0006	0.7931	0.0006
milk	pickled plum	6.06	0.13	6.06	0.03	0.51	0.9648	0.0003	0.2864	0.0003
pickled plum	cakes	0.13	0.14	0.19	0.94	0.11	0.0003	0.9437	0.0247	0.0003
radish	burdock	1.99	0.96	2.21	0.57	0.00	0.4947	0.6200	0.0003	0.0003

The positions of the nutrient content vector x and the standardized vector z for the top-ranking combination, bananas and peanuts, and the bottom-ranking combination, radishes and burdock (which is not an efficient combination for promoting the intake of calcium Ca, niacin, and vitamin B6), are shown in the scatter plot below.

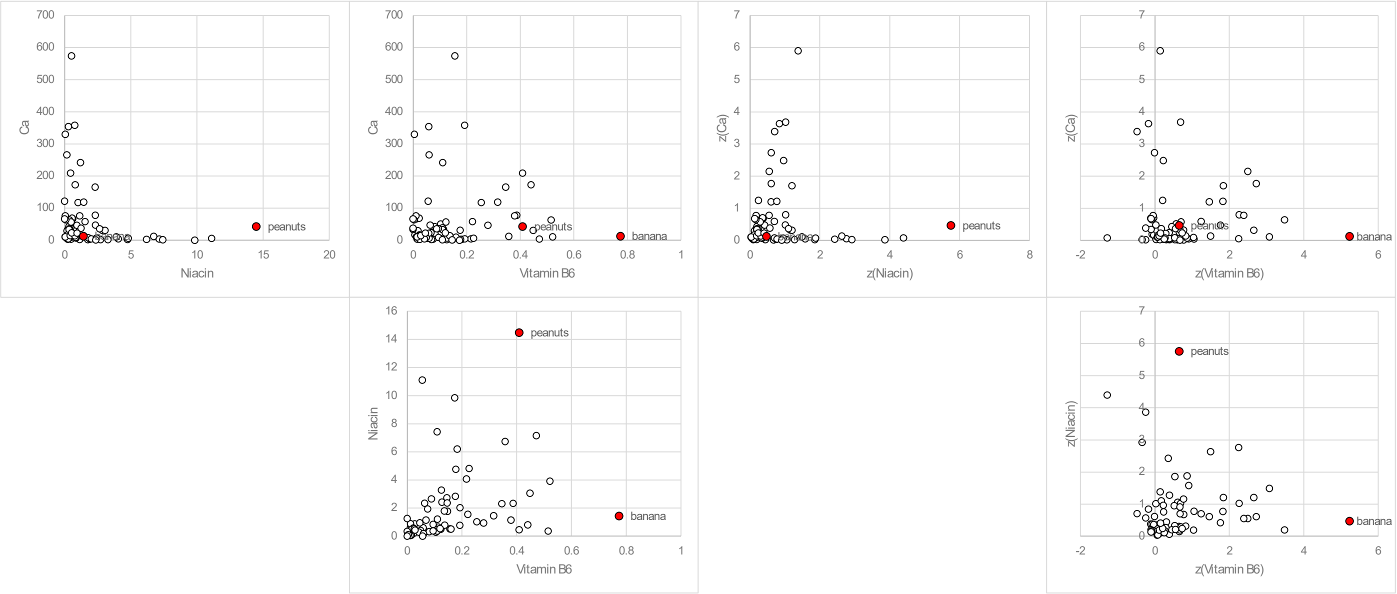

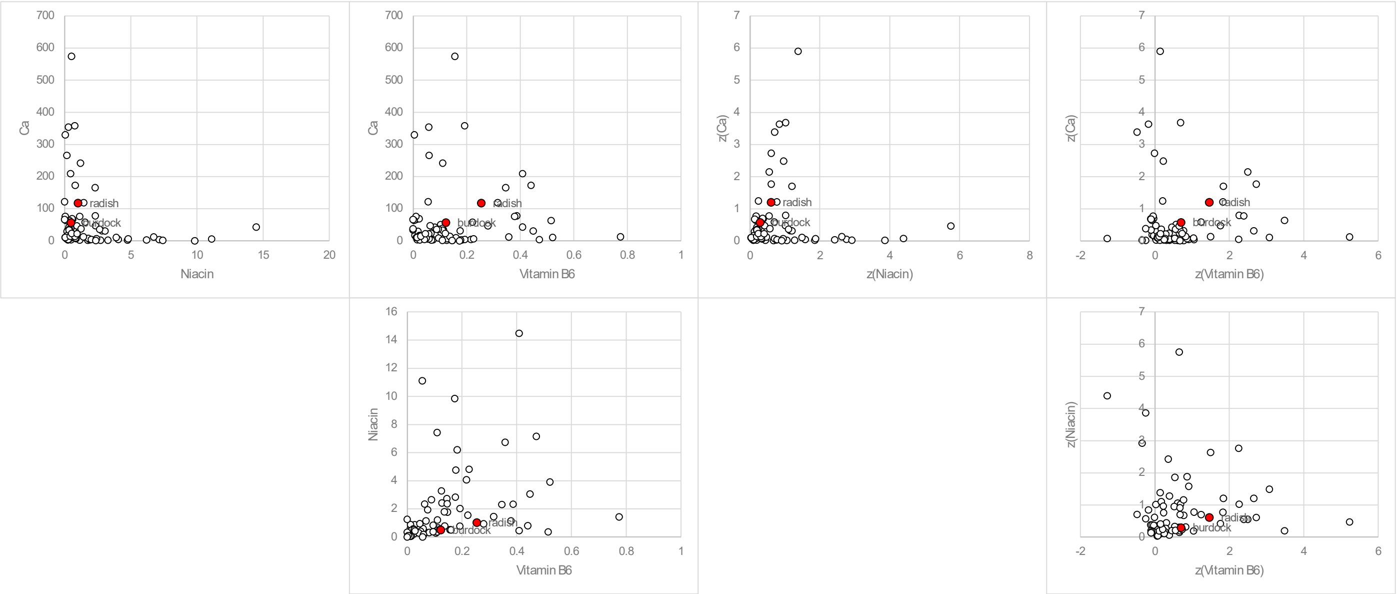

Note S3. Data wrangling of the Cookpad dataset:

To estimate the complementarity in recipes, we utilized the ingredient data from recipes included in the Cookpad dataset. The Cookpad data used in this article includes 1,715,595 recipes posted by a total of 158,398 users in Japan between around the year 2000 and 2014. Since the ingredient information for each recipe in the data is in the form of free-text data provided by the contributors, data cleaning was performed. As a result, the number of recipes that could be identified as using at least one of the 95 food items analyzed in this paper is 1,533,260 (89.4%) out of the total of 1,715,595. The data cleaning procedure is as follows.

1. From the data of ingredients in 1,715,595 recipes, duplicates were removed, yielding 1,117,475 unique texts representing ingredients.
2. For each of these 1,117,475 ingredient texts, the frequency of occurrence in the database was calculated, identifying 200,998 ingredient texts with a frequency of two or more.
3. Each of these 200,998 ingredient texts was matched to one of the 95 food items in the FFQ analyzed in this paper, resulting in 56,091 ingredient texts corresponding to the analyzed food items.
4. A total of 1,533,260 recipes incorporating the ingredients represented by these 56,091 texts were identified.

Note that in recipe ingredients, for instance, text data representing potatoes can appear in various forms, including hiragana, katakana, kanji, combinations of these, as well as synonyms such as "jagaimo" and "bareisho" in Japanese. While ingredient identification was not performed for 916,477 (82.0%) text entries with a frequency of less than two occurrences out of the total 1,117,475 entries, the identification of 200,998 (18.0%) entries with a frequency of two or more made it possible to determine that the analyzed ingredients were used in nearly 90% of recipes. Thus, sufficient coverage has been achieved.

In the text data cleaning, the problem of homonyms has not been completely eliminated. For example, if the ingredient name is entered as 'sake' in Japanese hiragana form, it is unclear whether it refers to fish (salmon) or alcohol (sake). In such cases, we uniformly treat it as fish (salmon).

Figure S2. The distribution of nutrient complementarity

(1) Female, 50≤age (a) Standard

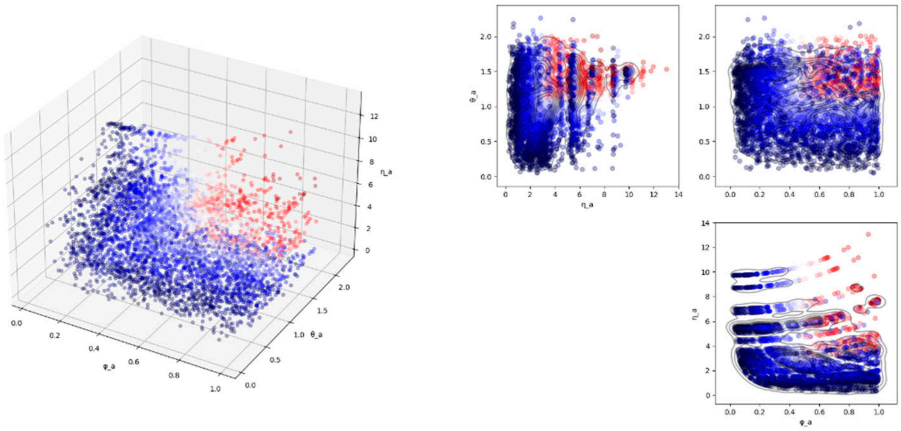

(1) Female, 50≤age (b) ¥100 equivalence

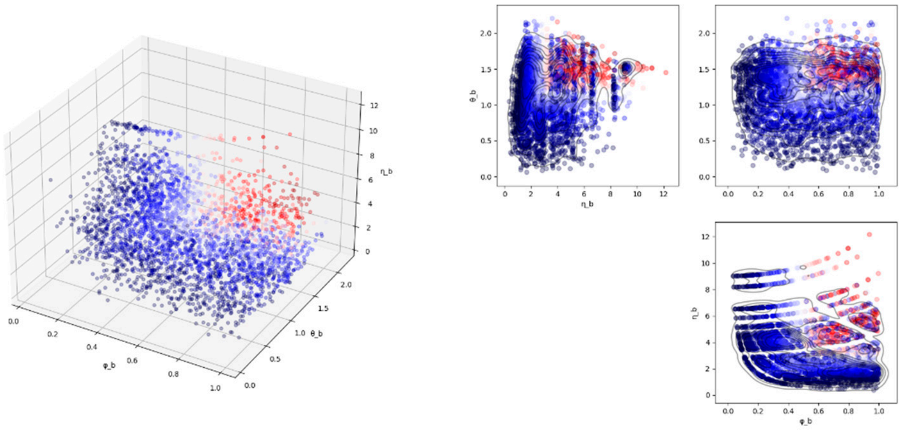

(2) Female, 20≤age<50 (a) Standard

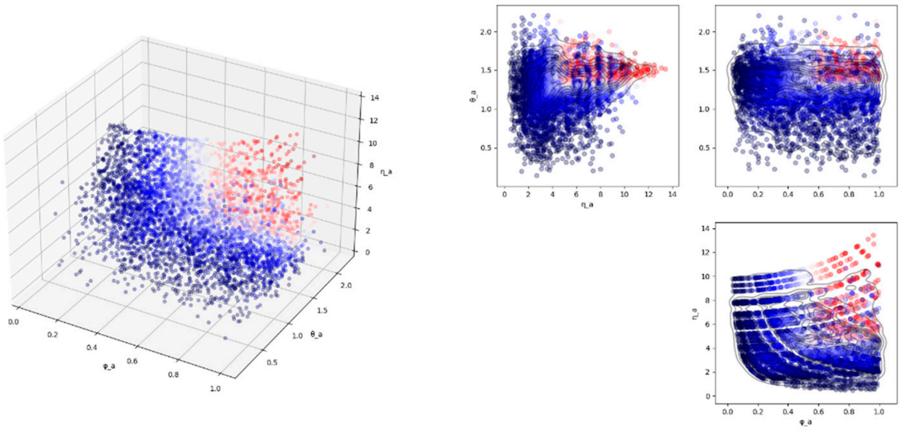

(2) Female, 20≤age<50 (b) ¥100 equivalence

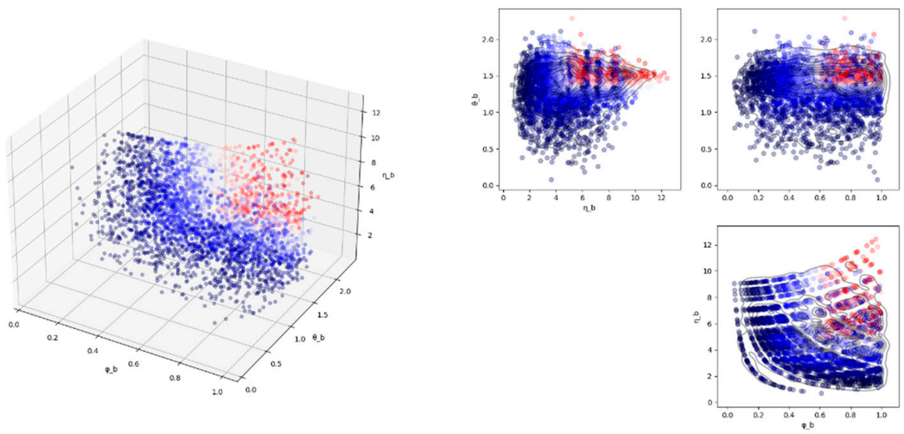

(3) Male, 50≤age (a) Standard

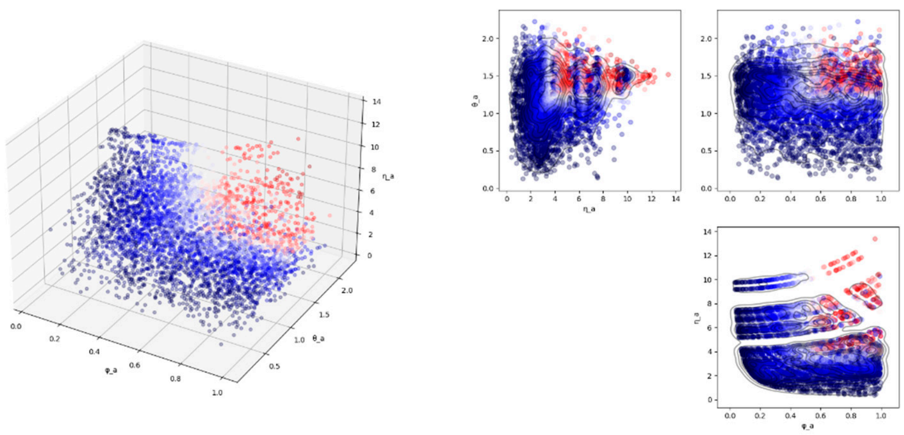

(3) Male, 50≤age (b) ¥100 equivalence

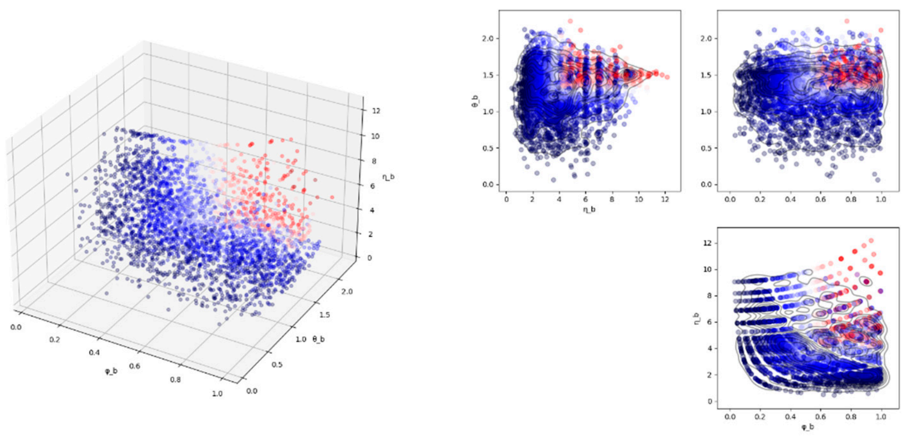

(4) Male, 20≤age<50 (a) Standard

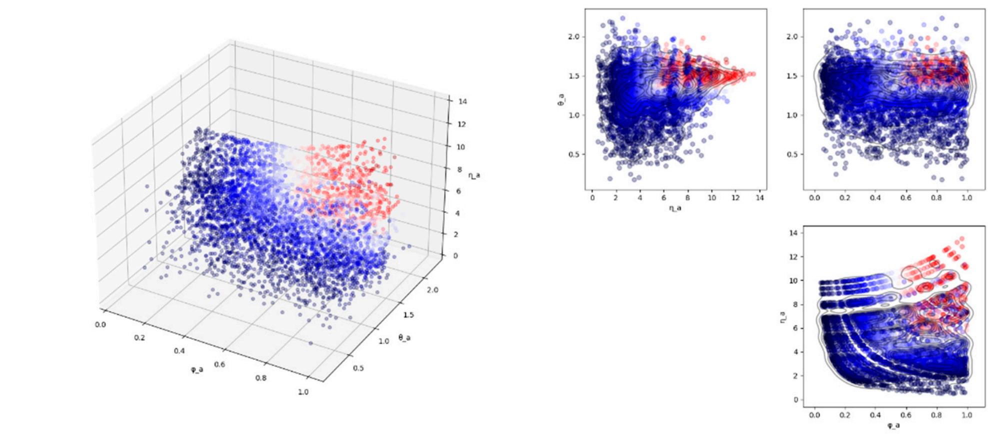

(4) Male, 20≤age<50 (b) ¥100 equivalence

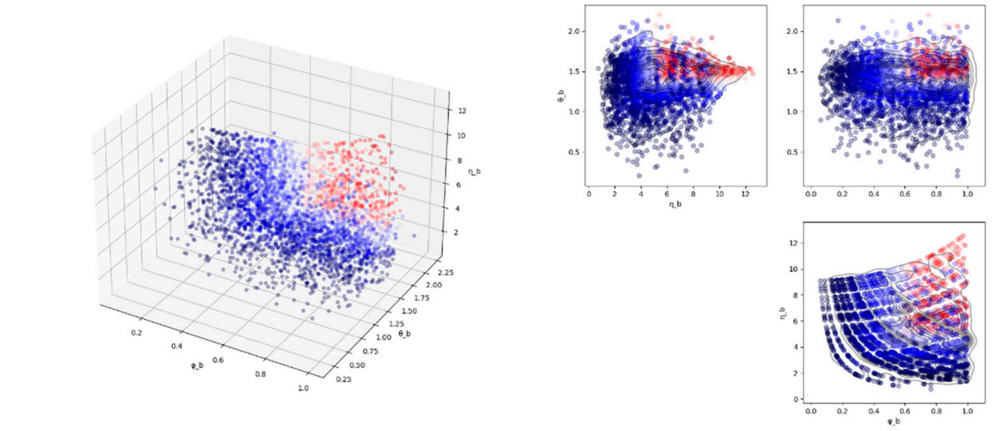

Figure S3. The overall view of the scatter plot of complementarity in habits and nutrient complementarity.

Due to the presence of outliers in the distribution of complementarity in preferences, which results in a clear cluster structure in the scatter plot, we will focus our analysis on the main cluster that includes more than 90% of the samples.

(1) Female, 50≤age (a) Standard	(1) Female, 50≤age (b) ¥100 equivalence
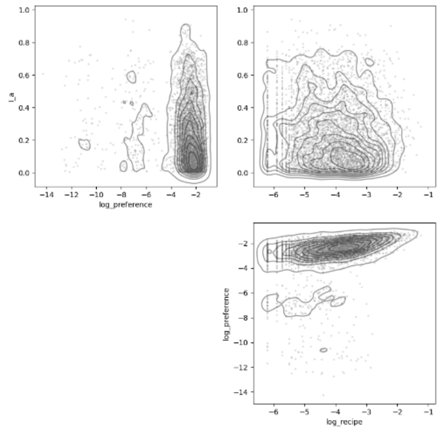	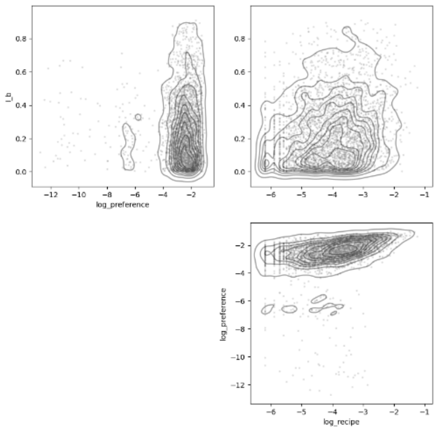

Figure S4. The sizes of $M(\tau)$ (blue lines) and $M(\tau) \cap R(\tau)$ (red lines)

(1) Female, 50≤age (a) Standard

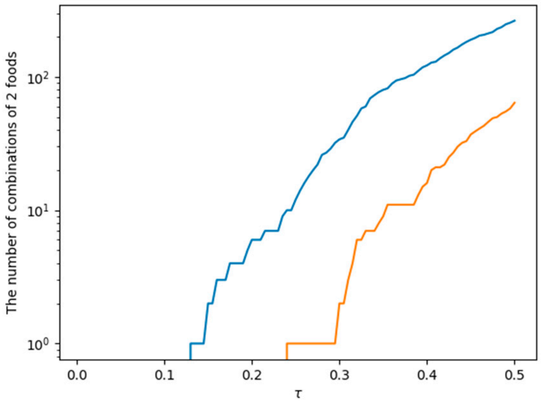

(1) Female, 50≤age (b) ¥100 equivalence

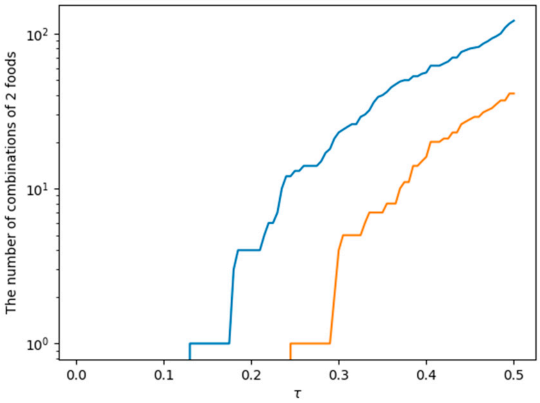

(2) Female, 20≤age<50 (a) Standard

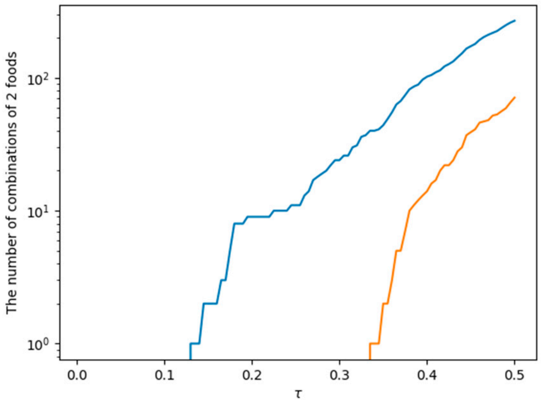

(2) Female, 20≤age<50 (b) ¥100 equivalence

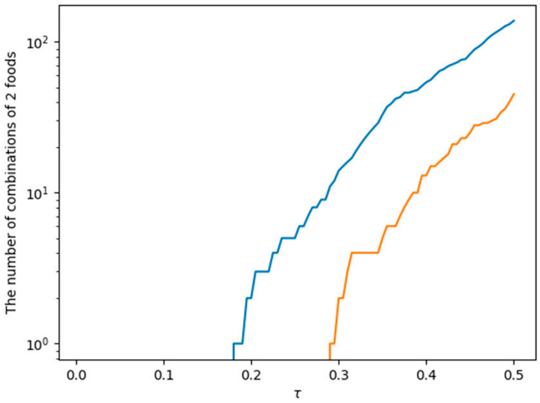

(3) Male, 50≤age (a) Standard

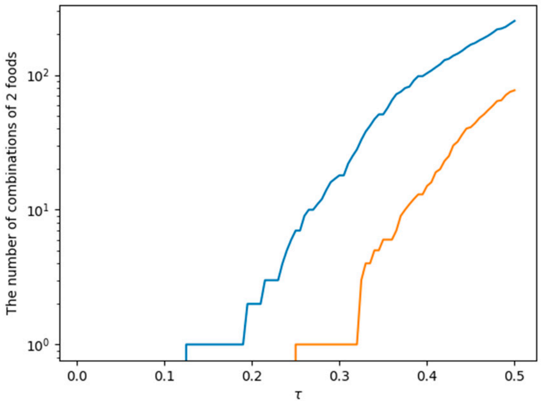

(3) Male, 50≤age (b) ¥100 equivalence

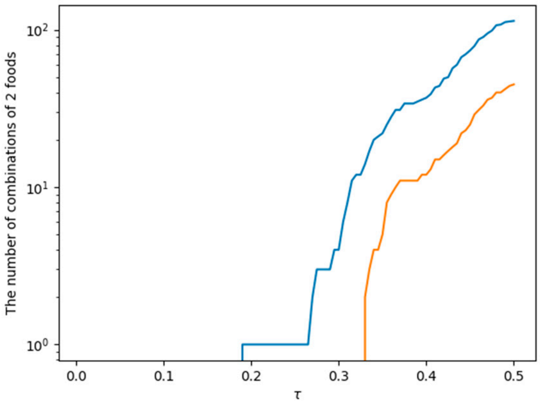

(4) Male, 20≤age<50 (a) Standard

(4) Male, 20≤age<50 (b) ¥100 equivalence

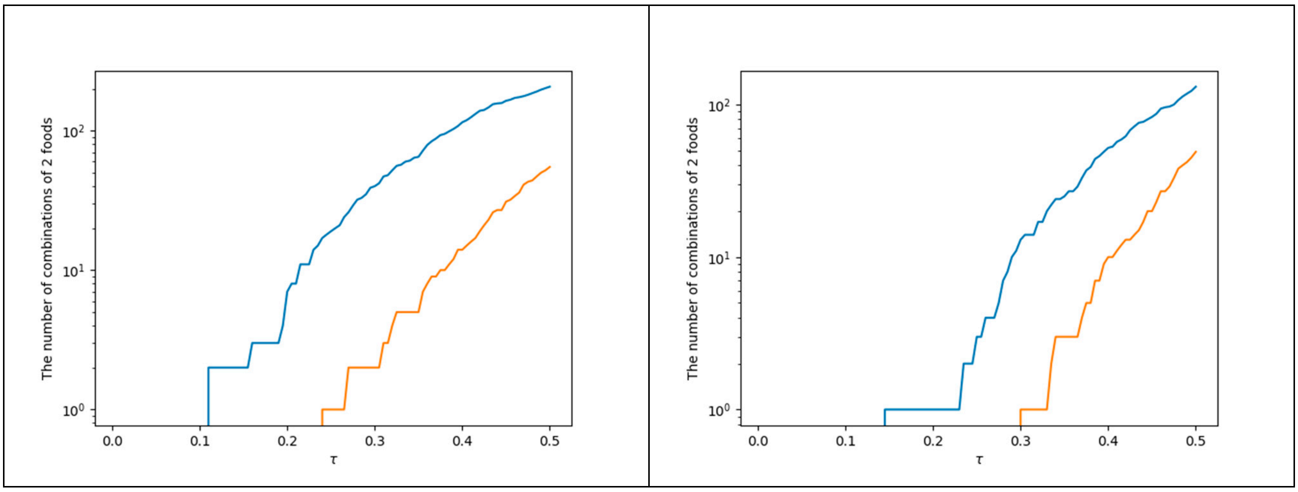
The number of combinations of 2 foods
τ
The number of combinations of 2 foods
τ

Figure S5. Nutrient intake in the sample data used for the analysis in this paper

The distribution of relative intake when DRI is set to 100 (exceptionally, for lipid, the deviation of intake from the lower limit of the target range for the percentage of energy). Blue represents males, and red represents females.

Aged 20 to 49	Aged 50 and above
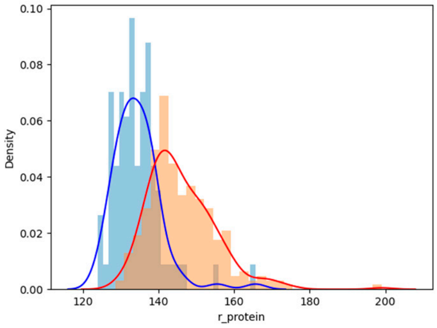	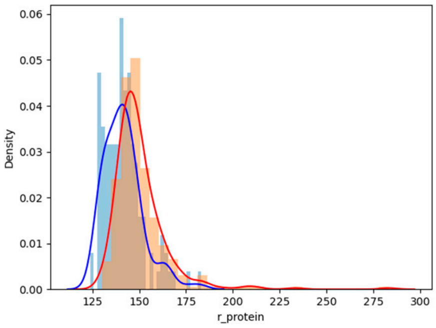
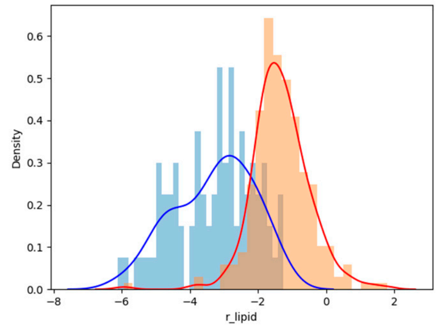	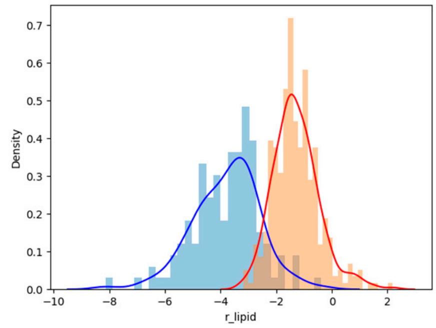
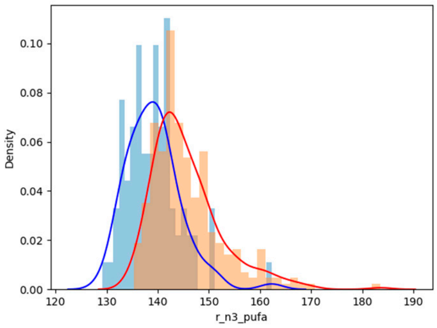	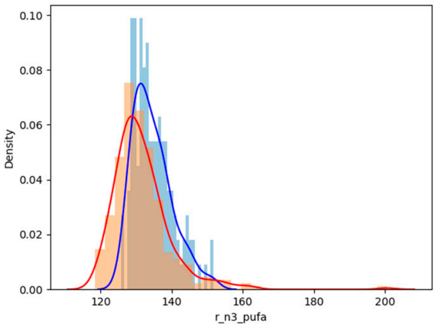
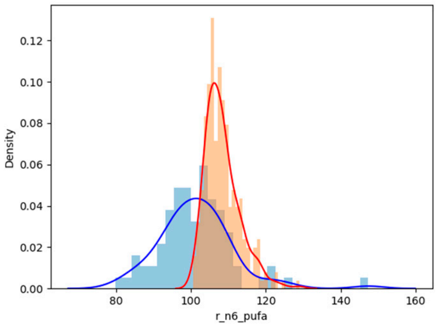	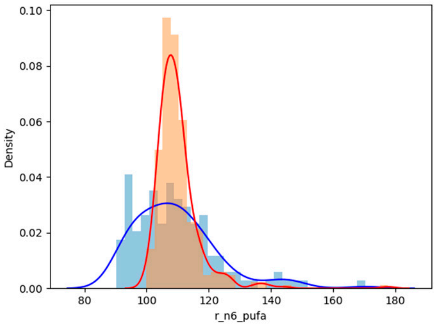

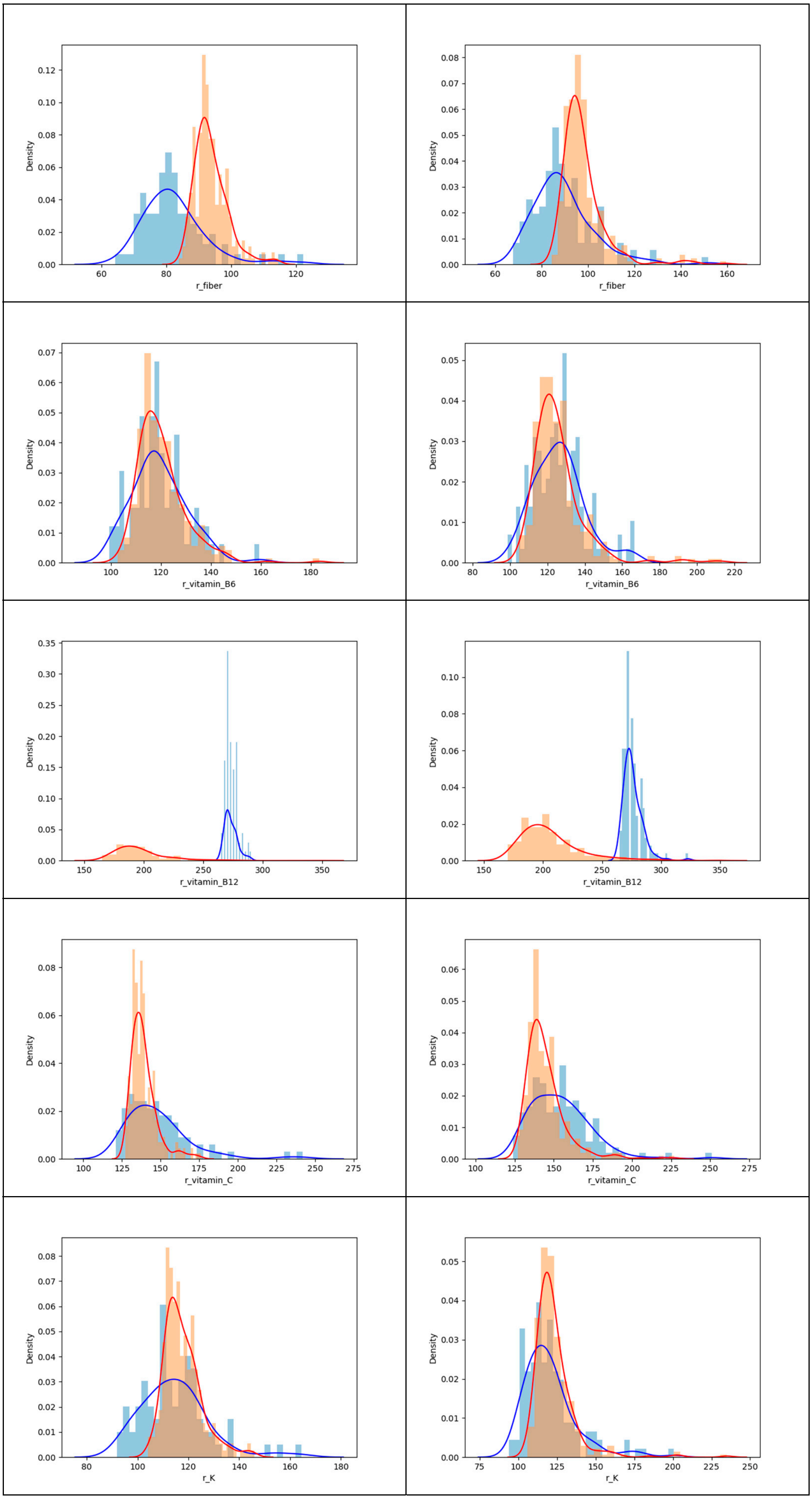
Density
r_fiber
Density
r_fiber
Density
r_vitamin_B6
Density
r_vitamin_B6
Density
r_vitamin_B12
Density
r_vitamin_B12
Density
r_vitamin_C
Density
r_vitamin_C
Density
r_K
Density
r_K

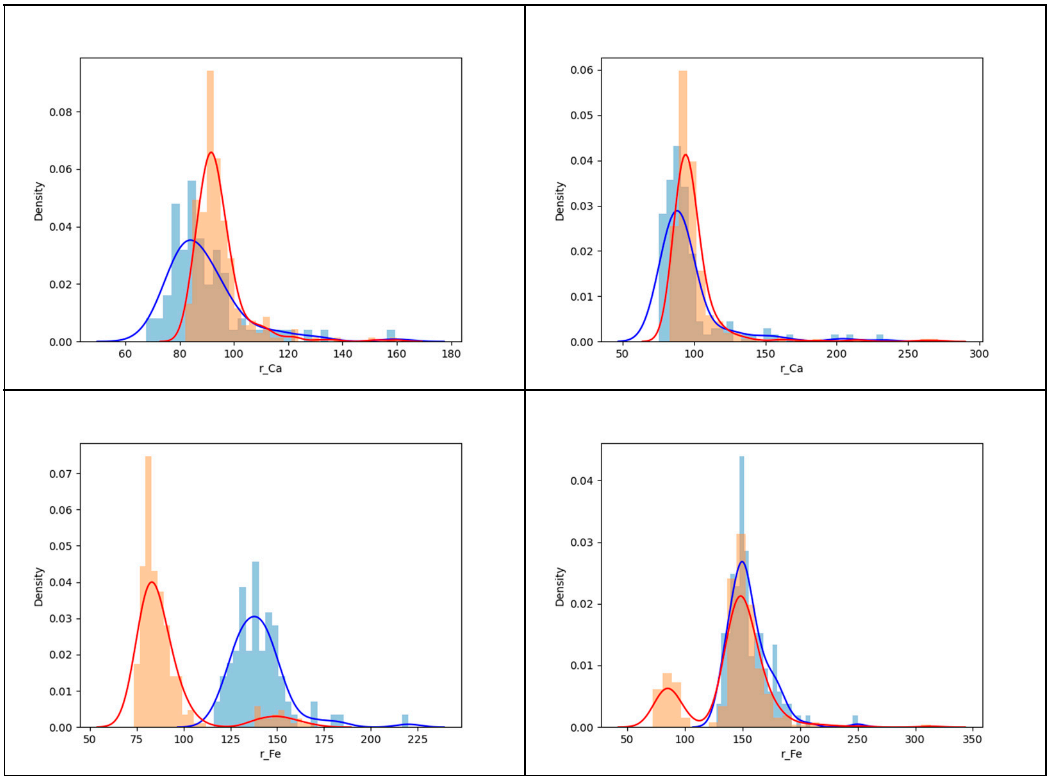
Density
r_Ca
Density
r_Ca
Density
r_Fe
Density
r_Fe

Table S1. Simulation analysis of the effects of FFQ measurement error on the estimation of complementarity in habits:

As a simulation, intake frequency data for food 1 and food 2 across 21 meals per week was generated 200 times using a multinomial distribution, $Multinomial(n = 21, p_1 = 0.06, p_2 = 0.03, p_3 = 0.01)$. Here, the probability of consuming only food 1 in a single meal was set at $p_1 = 0.06$, the probability of consuming only food 2 at $p_2 = 0.03$, and the probability of consuming both food 1 and food 2 simultaneously at $p_3 = 0.01$. The intake frequency of each food item was then converted into hierarchical data as per the FFQ questionnaire. For each food item, response bias was incorporated, with a probability q that the true value would be missclassifieded into one level higher or lower within the hierarchy. The correlation coefficient of events where biases occurred for food 1 and food 2 was denoted as ρ. For each combination of $q = (0.05, 0.10, 0.15, 0.20, 0.25)$ and $\rho = (0, 0.1, 0.2, 0.3, 0.4, 0.5)$, simulation data with a sample size of 200 was generated, and the probability p_3 of simultaneous intake was estimated 100 times.

(a) Upward bias in the responses to the frequency stratum of the two food items (true value is $p_{habit} = p_3 = 0.01$)

q	ρ	mean	std	min	10%	25%	50%	75%	90%	max
0.05	0.0	0.010	0.004	0.000	0.005	0.007	0.010	0.013	0.017	0.021
0.05	0.1	0.011	0.004	0.000	0.006	0.008	0.012	0.014	0.018	0.023
0.05	0.2	0.012	0.004	0.003	0.007	0.009	0.012	0.015	0.018	0.023
0.05	0.3	0.013	0.004	0.003	0.007	0.009	0.012	0.016	0.019	0.024
0.05	0.4	0.013	0.004	0.003	0.008	0.011	0.014	0.016	0.019	0.022
0.05	0.5	0.014	0.005	0.003	0.009	0.011	0.013	0.017	0.019	0.028
0.10	0.0	0.011	0.005	0.002	0.007	0.008	0.011	0.014	0.018	0.025
0.10	0.1	0.012	0.004	0.000	0.007	0.010	0.012	0.015	0.018	0.025
0.10	0.2	0.013	0.005	0.003	0.007	0.010	0.014	0.016	0.019	0.025
0.10	0.3	0.014	0.004	0.003	0.010	0.012	0.014	0.017	0.019	0.025
0.10	0.4	0.016	0.004	0.006	0.011	0.013	0.015	0.018	0.021	0.025
0.10	0.5	0.017	0.004	0.007	0.012	0.014	0.017	0.021	0.022	0.025
0.15	0.0	0.013	0.004	0.002	0.007	0.010	0.012	0.015	0.018	0.023
0.15	0.1	0.014	0.004	0.003	0.009	0.010	0.014	0.017	0.019	0.025
0.15	0.2	0.015	0.004	0.006	0.009	0.012	0.015	0.018	0.021	0.027
0.15	0.3	0.017	0.005	0.008	0.011	0.013	0.016	0.020	0.024	0.028
0.15	0.4	0.018	0.005	0.008	0.013	0.014	0.017	0.021	0.025	0.029
0.15	0.5	0.019	0.004	0.006	0.013	0.016	0.019	0.023	0.025	0.030
0.20	0.0	0.013	0.005	0.002	0.008	0.011	0.013	0.016	0.019	0.030
0.20	0.1	0.015	0.004	0.006	0.009	0.013	0.015	0.018	0.021	0.029
0.20	0.2	0.017	0.005	0.005	0.011	0.014	0.017	0.021	0.024	0.027
0.20	0.3	0.019	0.005	0.009	0.013	0.015	0.019	0.022	0.025	0.032
0.20	0.4	0.021	0.005	0.010	0.015	0.017	0.020	0.024	0.027	0.033
0.20	0.5	0.023	0.004	0.012	0.017	0.020	0.023	0.025	0.029	0.035
0.25	0.0	0.015	0.005	0.007	0.009	0.012	0.015	0.018	0.021	0.028
0.25	0.1	0.018	0.005	0.003	0.013	0.014	0.018	0.021	0.024	0.029
0.25	0.2	0.019	0.005	0.005	0.013	0.016	0.019	0.022	0.026	0.031
0.25	0.3	0.021	0.005	0.011	0.015	0.018	0.021	0.025	0.027	0.034
0.25	0.4	0.024	0.005	0.012	0.019	0.021	0.024	0.027	0.030	0.039
0.25	0.5	0.026	0.005	0.016	0.020	0.022	0.025	0.029	0.032	0.039

(b) Downward bias in the responses to the frequency stratum of the two food items (true value is $p_3 = p_{habit} = 0.01$)

q	ϱ	mean	std	min	10%	25%	50%	75%	90%	max
0.05	0.0	0.010	0.004	0.000	0.006	0.007	0.010	0.012	0.015	0.021
0.05	0.1	0.010	0.004	0.001	0.006	0.007	0.010	0.013	0.016	0.020
0.05	0.2	0.010	0.004	0.000	0.005	0.008	0.010	0.013	0.016	0.020
0.05	0.3	0.011	0.004	0.001	0.006	0.008	0.011	0.013	0.016	0.020
0.05	0.4	0.011	0.004	0.003	0.006	0.008	0.011	0.014	0.016	0.021
0.05	0.5	0.011	0.004	0.002	0.006	0.008	0.011	0.014	0.016	0.021
0.10	0.0	0.009	0.004	0.001	0.004	0.006	0.009	0.012	0.015	0.019
0.10	0.1	0.010	0.004	0.000	0.005	0.008	0.009	0.012	0.015	0.020
0.10	0.2	0.010	0.004	0.002	0.005	0.007	0.010	0.012	0.015	0.022
0.10	0.3	0.011	0.004	0.001	0.005	0.008	0.010	0.013	0.016	0.022
0.10	0.4	0.011	0.004	0.002	0.007	0.008	0.011	0.014	0.016	0.022
0.10	0.5	0.011	0.004	0.001	0.007	0.008	0.012	0.014	0.017	0.022
0.15	0.0	0.009	0.004	0.000	0.005	0.007	0.009	0.011	0.014	0.018
0.15	0.1	0.010	0.004	0.000	0.005	0.007	0.010	0.013	0.015	0.021
0.15	0.2	0.010	0.004	0.002	0.006	0.008	0.010	0.013	0.015	0.018
0.15	0.3	0.010	0.003	0.002	0.006	0.008	0.010	0.013	0.015	0.019
0.15	0.4	0.011	0.004	0.001	0.006	0.009	0.011	0.014	0.015	0.020
0.15	0.5	0.012	0.003	0.003	0.007	0.009	0.012	0.014	0.015	0.021
0.20	0.0	0.009	0.004	0.000	0.004	0.006	0.009	0.011	0.014	0.018
0.20	0.1	0.010	0.004	0.002	0.005	0.008	0.010	0.012	0.014	0.018
0.20	0.2	0.010	0.004	0.004	0.005	0.007	0.010	0.013	0.014	0.020
0.20	0.3	0.010	0.003	0.003	0.006	0.008	0.010	0.012	0.015	0.018
0.20	0.4	0.011	0.003	0.000	0.007	0.008	0.011	0.013	0.015	0.020
0.20	0.5	0.012	0.004	0.000	0.008	0.009	0.012	0.014	0.017	0.020
0.25	0.0	0.009	0.003	0.002	0.005	0.006	0.008	0.011	0.013	0.018
0.25	0.1	0.009	0.003	0.002	0.006	0.007	0.009	0.011	0.014	0.018
0.25	0.2	0.010	0.003	0.001	0.006	0.008	0.010	0.012	0.015	0.019
0.25	0.3	0.010	0.003	0.002	0.006	0.008	0.011	0.013	0.015	0.019
0.25	0.4	0.011	0.003	0.003	0.007	0.009	0.011	0.013	0.016	0.018
0.25	0.5	0.012	0.003	0.006	0.008	0.009	0.011	0.014	0.015	0.019

(c) Downward bias for food1 and upward bias for food2 in the responses to the frequency stratum of the two food items (true value is $p_3 = p_{habit} = 0.01$)

q	ϱ	mean	std	min	10%	25%	50%	75%	90%	max
0.05	0.0	0.010	0.004	0.000	0.004	0.007	0.009	0.012	0.014	0.019
0.05	0.1	0.009	0.004	0.000	0.004	0.006	0.009	0.011	0.015	0.020
0.05	0.2	0.009	0.004	0.000	0.004	0.006	0.008	0.011	0.014	0.021
0.05	0.3	0.008	0.004	0.000	0.003	0.005	0.008	0.010	0.013	0.021
0.05	0.4	0.008	0.004	0.000	0.003	0.005	0.008	0.010	0.014	0.020
0.05	0.5	0.007	0.004	0.000	0.002	0.004	0.007	0.009	0.013	0.019
0.10	0.0	0.009	0.004	0.000	0.005	0.006	0.009	0.012	0.014	0.021
0.10	0.1	0.008	0.004	0.000	0.003	0.006	0.008	0.011	0.013	0.020
0.10	0.2	0.007	0.004	0.000	0.002	0.004	0.007	0.010	0.013	0.018
0.10	0.3	0.006	0.004	0.000	0.002	0.004	0.006	0.008	0.011	0.020
0.10	0.4	0.005	0.004	0.000	0.000	0.002	0.005	0.008	0.011	0.017
0.10	0.5	0.005	0.004	0.000	0.000	0.001	0.004	0.007	0.010	0.016
0.15	0.0	0.008	0.004	0.000	0.002	0.005	0.007	0.011	0.013	0.020
0.15	0.1	0.006	0.004	0.000	0.001	0.002	0.006	0.009	0.011	0.017
0.15	0.2	0.006	0.004	0.000	0.001	0.003	0.005	0.008	0.011	0.015
0.15	0.3	0.004	0.003	0.000	0.000	0.001	0.003	0.007	0.009	0.014
0.15	0.4	0.004	0.003	0.000	0.000	0.000	0.003	0.006	0.009	0.015
0.15	0.5	0.003	0.003	0.000	0.000	0.000	0.002	0.004	0.006	0.011
0.20	0.0	0.006	0.004	0.000	0.002	0.004	0.006	0.009	0.012	0.018
0.20	0.1	0.005	0.004	0.000	0.000	0.002	0.004	0.008	0.011	0.016
0.20	0.2	0.004	0.004	0.000	0.000	0.001	0.003	0.006	0.009	0.014
0.20	0.3	0.003	0.004	0.000	0.000	0.001	0.002	0.006	0.007	0.017
0.20	0.4	0.002	0.003	0.000	0.000	0.000	0.002	0.004	0.006	0.015
0.20	0.5	0.002	0.002	0.000	0.000	0.000	0.001	0.003	0.005	0.011
0.25	0.0	0.005	0.004	0.000	0.000	0.002	0.005	0.007	0.011	0.017
0.25	0.1	0.004	0.003	0.000	0.000	0.001	0.003	0.006	0.008	0.016
0.25	0.2	0.003	0.003	0.000	0.000	0.000	0.002	0.005	0.008	0.013
0.25	0.3	0.003	0.003	0.000	0.000	0.000	0.002	0.004	0.007	0.015
0.25	0.4	0.001	0.002	0.000	0.000	0.000	0.000	0.002	0.005	0.012
0.25	0.5	0.001	0.002	0.000	0.000	0.000	0.000	0.002	0.004	0.008
